# Abnormal grey matter structural changes in patients with end-stage kidney disease and mild cognitive impairment: correlations with clinical features

**DOI:** 10.1007/s11011-023-01293-5

**Published:** 2023-09-30

**Authors:** Huijie Yuan, Zhaoyao Luo, Wen Gu, Shaohui Ma, Guangyu Li, Dun Ding, Xueying Ma, Peng Li, Jing Yang, Xiaoling Xu, Junya Mu, Ming Zhang

**Affiliations:** 1 Department of Medical Imaging, First Affiliated Hospital of Xi’an Jiaotong University, Xi’an, Shaanxi 710061 China; 2 Department of Medical Imaging, Second Affiliated Hospital of Xi’an Jiaotong University, Xi’an, Shaanxi China; 3 Department of Medical Imaging, the Affiliated Hospital of Inner Mongolia Medical University, Hohhot, Inner Mongolia China

**Keywords:** End-stage kidney disease, Grey matter volume, Mild cognitive impairment, Magnetic resonance imaging, Voxel-based morphometry

## Abstract

End-stage kidney disease and mild cognitive impairment (ESKD-MCI) affect the quality of life and long-term treatment outcomes of patients affected by these diseases. Clarifying the morphological changes from brain injuries in ESKD-MCI and their relationship with clinical features is helpful for the early identification and intervention of MCI before it progresses to irreversible dementia. This study gathered data from 23 patients with ESKD-MCI, 24 patients with ESKD and non-cognitive impairment (NCI), and 27 health controls (HCs). Structural magnetic resonance studies, cognitive assessments, and general clinical data were collected from all participants. Voxel-based morphometry analysis was performed to compare grey matter (GM) volume differences between the groups. The patients’ GM maps and clinical features were subjected to univariate regression to check for possible correlations. Patients with ESKD-MCI displayed significantly more impairments in multiple cognitive domains, including global cognition, visuospatial and executive function, and memory, compared to patients with ESKD-NCI. Using a more liberal threshold (*P* < 0.001, uncorrected), we found that compared to patients with ESKD-NCI, patients with ESKD-MCI exhibited clusters of regions with lower GM volumes, including the right hippocampus (HIP), parahippocampal gyrus (PHG), Rolandic operculum, and supramarginal gyrus. The volumes of the right HIP and PHG were negatively correlated with serum calcium levels. ESKD-MCI was associated with a subtle volume reduction of GM in several brain areas known to be involved in memory, language, and auditory information processing. We speculate that these slight morphometric impairments may be associated with disturbed calcium metabolism.

## Introduction

End-stage kidney disease (ESKD) is a progressive, incurable, and systemic disease (Collaboration et al., 2020). Although continuous advances in renal replacement therapy have effectively prolonged the survival of patients with ESKD, cognitive impairment (CI) is an important factor affecting the quality of life and prognosis of these patients (Kurella Tamura and Yaffe 2011). Mild cognitive impairment (MCI) can be identified in approximately 16–60% of patients with ESKD, which is two or three times higher than that in the age-matched general population (Viggiano et al. 2020a). Identifying MCI early, and exploring its underlying neuropathological mechanisms, may allow for early intervention, before irreversible damage occurs in patients with ESKD.

Recently, the role of imaging features and biological markers in the diagnosis of MCI has garnered increased interest (Albert et al. 2011). With the popularity and development of neuroimaging techniques, including magnetic resonance imaging (MRI) and positron emission tomography, patients with CI who may have been misdiagnosed or neglected can be diagnosed earlier and more accurately (Ellis et al. 2011). Structural MRI (sMRI) is inherently stable, radiation-free, non-invasive, and easy to perform. In the last decade, many studies have investigated macrostructural features of the brain in patients with ESKD using sMRI, with inconsistent results. However, a more consistent finding was that patients with ESKD had significantly abnormal grey matter (GM) volumes in areas associated with advanced cognitive functions, including attention and executive function (Qiu et al. 2014; Li et al. 2018; Wang et al. 2022), memory (Chiu et al. 2019; Gu et al. 2021; Jin et al. 2020; Li et al. 2018; Qiu et al. 2014; Wang et al. 2022), and auditory processing (Chai et al. 2015; Li et al. 2018; Wang et al. 2022) compared to healthy controls (HCs). The pattern of alterations in brain morphology in patients with ESKD and MCI remains unclear. Additionally, it should be noted that many of these studies included patients with ESKD who were on dialysis, whereas dialysis treatment itself had an impact on cognition, and did not stratify the degree of cognitive decline.

Among the various sMRI analysis techniques, voxel-based morphometry (VBM) has become popular because of its simplicity in operation and relatively stable and reliable results. VBM is an automated technique that calculates volume differences between groups by performing statistical tests for all voxels in the image. It can also be used to regress across voxels to assess the neuroanatomical correlates of cognitive deficits and clinical features (Whitwell 2009).

The purpose of the present study was to utilize VBM for comparing brain GM volumes among patients with ESKD and MCI, patients with ESKD and non-cognitive impairment (NCI) before the initiation of dialysis, as well as HCs. Additionally, we aimed to explore whether a relationship existed between morphological patterns and clinical features (cognitive function and biochemical blood indicators) in patients with ESKD and MCI.

## Materials and methods

### Participants and enrollment criteria

**Fig. 1:**
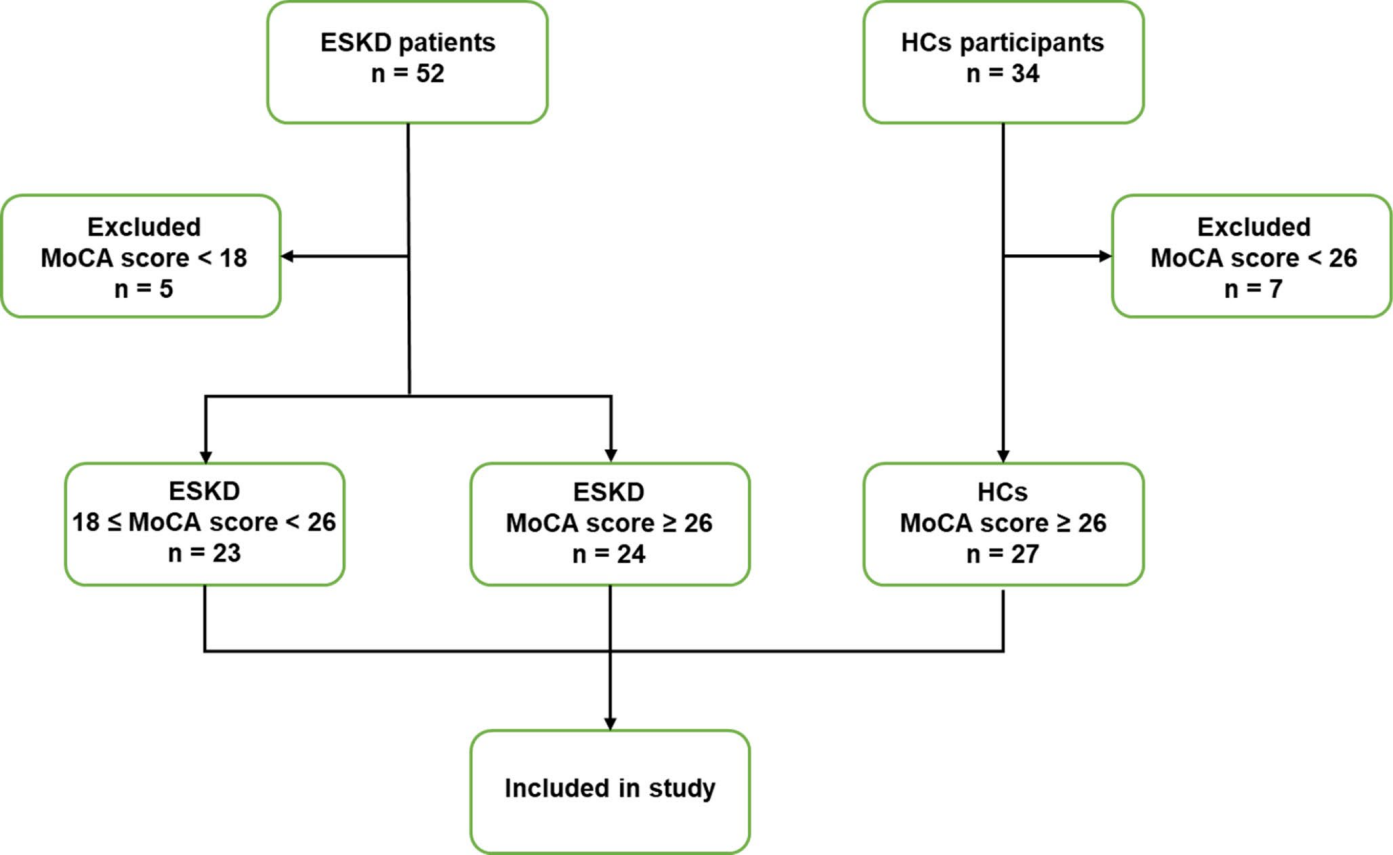
The flowchart shows the selection criteria for patients with end-stage kidney disease and mild cognitive impairment (ESKD-MCI), ESKD and non-cognitive impairment (ESKD-NCI), and healthy controls (HCs) in the study. MoCA: Montreal cognitive assessment

This study protocol was registered at ClinicalTrials.gov (NCT03961724, https://clinicaltrials.gov/ct2/show/NCT03961724) and received local ethics committee approval (XJTU1AF-CRF-2018-006). Figure 1 presents a flowchart depicting the selection criteria. Patients with ESKD who had never received dialysis were recruited from the Department of Nephrology at the local hospital. HCs with no history of neurologic, psychiatric, or other major medical illnesses were recruited by posting advertisements in the local community, and those with Montreal Cognitive Assessment (MoCA) scores < 26 were excluded.

The inclusion criteria for patients with ESKD were as follows: (1) aged 18–50 years; (2) estimated glomerular filtration rate (eGFR) < 15 mL/min/1.73m^2^; (3) not yet on dialysis or recipient of a kidney transplant. The exclusion criteria were as follows: (1) history of neurological or psychiatric disease, organic brain disease, or diabetes; (2) history of drug abuse or alcohol addiction; (3) macroscopic brain T2-visible lesions on MRI scans; (4) previously confirmed dementia, including Alzheimer’s disease (AD), frontotemporal dementia, or Parkinson’s disease dementia; (5) MoCA scores < 18; (6) contraindications to MRI examination; and (7) inability to cooperate with the completion of the MRI examination or cognitive function assessments.

### Cognitive function assessments and biochemical blood tests

All subjects completed a series of standardized cognitive function assessments before undergoing the MRI scan. The Montreal Cognitive Assessment (MoCA) test, Beijing version, was used for assessing global cognitive function. It included visuospatial/executive function (5 points), naming (3 points), attention (6 points), language (3 points), abstraction (2 points), delayed recall(5 points) and orientation(6 points), with a total possible score of 30 points. If the education level was ≤ 12 years, 1 point was added to reduce educational bias (Nasreddine et al. 2005). The Auditory Verbal Learning Test, Huashan version (AVLT-H), was used for evaluating memory. It included an immediate recall total score (IR-S), short-term delayed recall score (SR-S), long-term delayed recall score (LR-S), and recognition score (REC-S) (Guo et al., 2009). The Trail Making Test part A (TMT-A) was used to evaluate processing speed (Bowie and Harvey 2006).

Biochemical blood tests were conducted for all patients with ESKD, including haemoglobin (g/L), haematocrit (%), blood urea nitrogen (BUN, mmol/L), serum creatinine (SCr, µmol/L), uric acid (µmol/L), cystatin C (CysC, mg/L), calcium (mmol/L), phosphorus (mmol/L), potassium (mmol/L), sodium (mmol/L), and parathyroid hormone (PTH, pg/mL), up to 24 h prior to the MRI scans.

### Groups

Patients with ESKD who met the inclusion criteria were divided into two groups: patients with ESKD and MCI (ESKD-MCI), and those with ESKD and NCI (ESKD-NCI). The former was defined as ESKD patients who met the 2003 International Working Group criteria for a diagnosis of MCI (Winblad et al. 2004), while the latter were those with no evidence of CI. The MoCA was specifically designed to detect MCI, using a cut-off score point of 25/26, with a sensitivity of 80–100% and specificity of 50–76% (Nasreddine et al. 2005; Lin et al. 2013). Patients with MoCA scores ≥ 18 and < 26 were classified as ESKD-MCI, while those with scores ≥ 26 were classified as ESKD-NCI.

### Image protocols

A 3 Tesla GE Excite MRI scanner (GE Medical Systems, Milwaukee, WI, USA) was employed to acquire images for all subjects. Structural image data for each participant were obtained using a T1-weighted three dimensional fast spoiled gradient echo sequence with the following parameters: 140 axial slices; slice thickness = 1.0 mm; echo time = 4.8 ms; repetition time = 10.8 ms; flip angle = 9°; no gap; matrix = 256 × 256; and field of view = 256 × 256 mm^2^.

### Image data processing

Structural image data were processed using the Computational Anatomy Toolbox 12 software (CAT12, http://www.neuro.uni-jena.de/cat/), an extension of Statistical Parametric Mapping 12 (SPM12, http://www.fil.ion.ucl.ac.uk/spm/software/spm12/), in MATLAB 2021a (MathWorks Inc., Natick, USA). Initially, we checked the sample homogeneity in CAT12 to identify poor-quality images and incorrect pre-processing. No abnormalities were observed in the acquired or pre-processed images. Subsequently, each participant’s images were reoriented to share the same anterior commissure point of origin and spatial orientation. SPM12 tissue probability maps were used for the initial spatial registration and segmentation. GM, white matter (WM), and cerebrospinal fluid data were obtained using spatial segmentation to calculate the overall tissue volume and total intracranial volume (TIV). Next, the standard Montreal Neurological Institute (MNI) template in CAT12 was used to normalize the standard space, with the Diffeomorphic Anatomical Registration through the Exponentiated Lie algebra (DARTEL) toolbox used for GM and WM. After completion of the pre-processing steps, quality checks were performed using the CAT12 toolbox to assess the homogeneity of the GM tissues. Finally, the modulated and normalized GM tissue segments were smoothed with an 8-mm full width at half maximum Gaussian filter.

### Statistical analysis

Intergroup differences in subjects’ demographics were analyzed using SPSS software (version 26.0; IBM Corp, Armonk, NY, USA). Additionally, differences in cognitive data between the groups were compared using one-way analysis of covariance (ANCOVA) adjusted for sex, age, and education level. A *p*-value < 0.05 indicated a significant difference. A statistical power analysis was performed on sample size.

Voxel-wise group comparisons (ESKD-MCI vs. HCs, ESKD-NCI vs. HCs, and ESKD-MCI vs. ESKD-NCI) of smoothed GM volumes were performed using two-sample *t*-tests within SPM 12, with age, sex, and TIV as covariates. Family-wise error (FWE) correction was applied for multiple comparison corrections with a threshold of *p* < 0.05. Meanwhile, an exploratory analysis was conducted using less conservative uncorrected thresholds of *p* < 0.001 for the entire brain.

Multiple univariate regression analyses were carried out using the SPM12 model design tool to access whether regional GM volume changes (ESKD-MCI vs. ESKD-NCI) were associated with clinical features (cognitive tests and biochemical blood indicators) in ESKD-MCI, with age, sex, education level, and TIV as covariates. These analyses were performed with a threshold of *p* < 0.001 and were uncorrected.

## Results

### Demographic data and clinical profiles

**Fig. 2:**
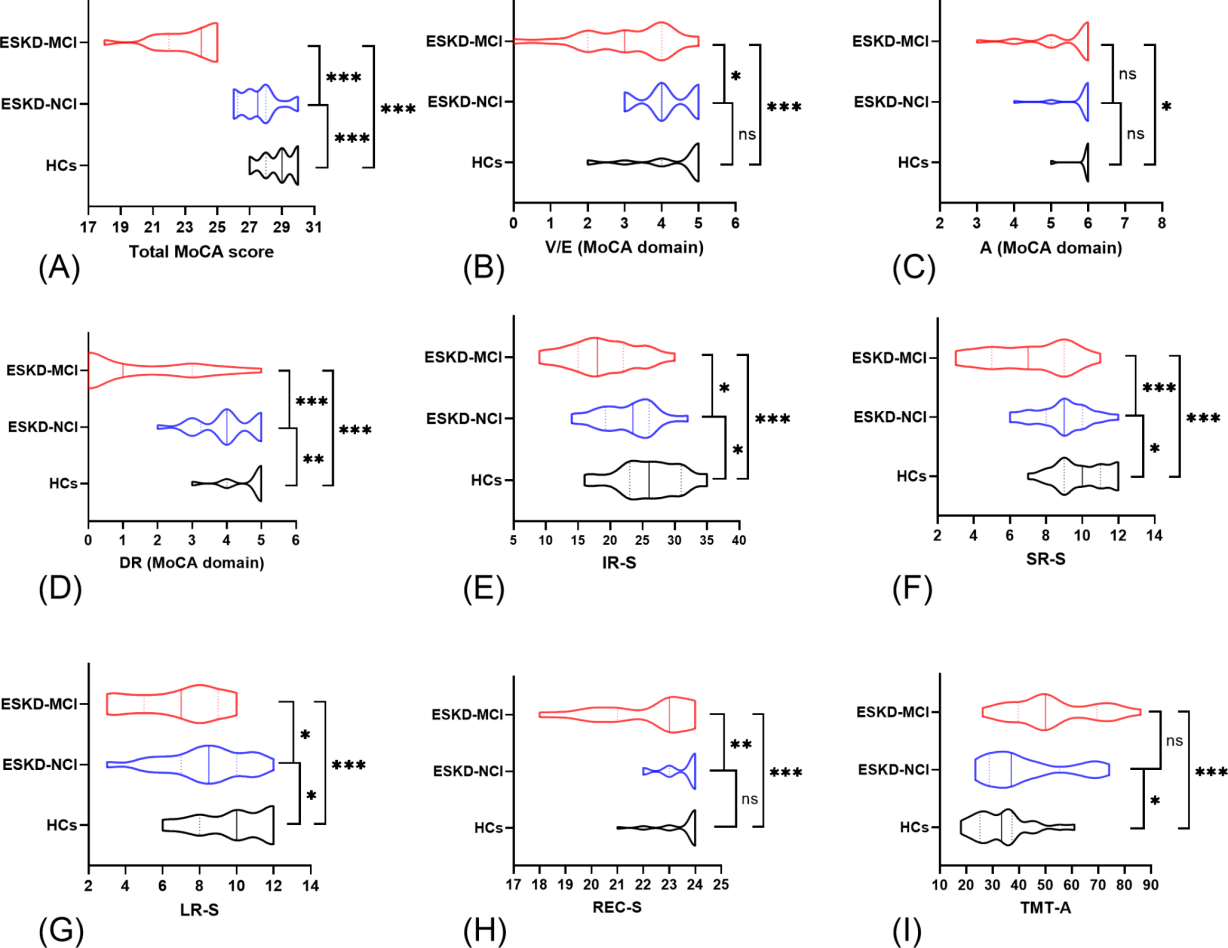
The violin plot depicts intergroup differences in cognitive function assessments between the patients with end-stage kidney disease and mild cognitive impairment (ESKD-MCI), those with ESKD and non-cognitive impairment (ESKD-NCI), and healthy controls (HCs), adjusted for age, sex, and education level MoCA, Montreal Cognitive Assessment; V/E, visuospatial/executive; A, attention; DR, delayed recall; IR-S, immediate recall total score; SR-S, short-term delayed recall score; LR-S, long-term delayed recall score; REC-S, recognition score; TMT-A, trail-making test, part A. * *p* ≤ 0.05; ** *p* ≤ 0.01; *** *p* ≤ 0.001

A total of 47 patients with ESKD, who had never received dialysis (23 ESKD-MCI, 24 ESKD-NCI) and 27 HCs were included in the present study. Intergroup differences in demographic data and clinical profiles are presented in Table 1. No significant differences were observed in pairwise comparisons among the three groups in terms of sex, age, or education level. Furthermore, no differences in the biochemical blood indicators were noted between the ESKD-MCI and ESKD-NCI groups. Group differences in cognitive function assessments, adjusted for age, sex, and education level, are detailed in Table 2 and illustrated in Fig. 2. Compared to HCs, the ESKD-MCI group showed poorer global cognitive function (*p* < 0.001, effect size = 3.64, two-sided α = 0.05, 1-β = 0.95, actual power = 100%), visuospatial/executive function, attention, memory, and processing speed, while compared to HCs, the ESKD-NCI group demonstrated poorer global cognitive function (*p* < 0.001, effect size = 1.07, two-sided α = 0.05, 1-β = 0.95, actual power = 96%), memory, and processing speed. When compared to the ESKD-NCI group, the ESKD-MCI group exhibited inferior global cognitive function (*p* < 0.001, effect size = 2.66, two-sided α = 0.05, 1-β = 0.95, actual power = 100%), visuospatial/executive function, and memory.

**Table 1:** Demographic data from patients with ESKD as well as HCs, and biochemical profiles of the patients

Variables	HCs (n = 27)	All Patients with ESKD (n = 47)	*P*- value	ESKD-MCI (n = 23)	ESKD-NCI (n = 24)	*P*-value for ESKD-MCI vs. HCs	*P*-value for ESKD-NCI vs. HCs	*P*-value for ESKD-MCI vs. ESKD-NCI
Demographics								
Male, n (%)	16 (59.26)	33 (70.21)	0.338^a^	14 (60.87)	19 (79.17)	0.908^a^	0.126^a^	0.170^a^
Age (years), mean ± SD	35.44 ± 8.48	35.04 ± 10.41	0.645^b^	35.70 ± 11.14	34.42 ± 9.86	0.928^c^	0.691^c^	0.679^c^
n (%)			0.757^d^			0.715^d^	1.000^d^	0.724^d^
< 45	23 (85.19)	38 (80.85)		18	20			
≥ 45	4 (14.81)	9 (19.15)		5	4			
Education (years), mean ± SD	12.74 ± 3.31	12.62 ± 3.13	0.758^b^	11.70 ± 2.58	13.50 ± 3.40	0.227^b^	0.523^b^	0.059^b^
Duration (months), median (IQR)	/	30	/	48	24	/	/	0.405^b^
Biochemical, mean ± SD								
Hemoglobin (g/L)	/	91.28 ± 16.16	/	91.04 ± 15.42	91.50 ± 17.16	/	/	0.924^c^
Hematocrit (%)	/	27.19 ± 5.60	/	27.64 ± 5.07	26.75 ± 6.14	/	/	0.590^c^
Calcium (mmol/L)	/	2.02 ± 0.21	/	1.98 ± 0.21	2.05 ± 0.21	/	/	0.088^b^
Phosphorus (mmol/L)	/	1.71 ± 0.53	/	1.75 ± 0.55	1.68 ± 0.51	/	/	0.675^c^
Potassium (mmol/L)	/	4.55 ± 0.68	/	4.69 ± 0.69	4.42 ± 0.65	/	/	0.166^c^
Sodium (mmol/L)	/	140.70 ± 3.45	/	141.17 ± 3.06	140.25 ± 3.79	/	/	0.364^c^
SCr (µmol/L)	/	812.02 ± 284.14	/	787.57 ± 238.75	835.46 ± 302.02	/	/	0.569^c^
BUN (mmol/L)	/	27.87 ± 9.43	/	27.79 ± 7.34	27.94 ± 11.24	/	/	0.955^c^
Uric acid (µmol/L)	/	482.82 ± 167.40	/	495.48 ± 188.94	470.70 ± 146.93	/	/	0.617^c^
CysC (mg/L)	/	4.00 ± 1.00	/	4.12 ± 1.17	3.89 ± 0.81	/	/	0.448^c^
PTH (pg/ml)	/	278.23 ± 183.36	/	292.87 ± 210.16	264.20 ± 156.78	/	/	0.815^b^

ESKD, end-stage kidney disease; HCs, healthy controls; ESKD-MCI, ESKD and mild cognitive impairment; ESKD-NCI, ESKD and non-cognitive impairment; SD, standard deviation; SCr, serum creatinine; BUN, blood urea nitrogen; CysC, cystatin C; PTH, parathormone

^a^Analyzed using chi-square test; ^b^Analyzed using Mann-Whitney U test; ^c^Analyzed using independent two-sample *t*-tests; ^d^Analyzed using Fisher’s exact test

**Table 2:** Cognitive assessments of patients with ESKD as well as HCs.

Variables	HCs (n = 27)	ESKD-MCI (n = 23)	ESKD-NCI (n = 24)	Adjusted the *p*-value for ESKD-MCI vs. HCs	Adjusted the *p*-value for ESKD-NCI vs. HCs	Adjusted the *p*-value for ESKD-MCI vs. ESKD-NCI
MoCA	28.81 ± 1.08	23.30 ± 1.85	27.54 ± 1.29	**< 0.001**	**< 0.001**	**< 0.001**
V/E	4.48 ± 0.94	3.13 ± 1.25	4.17 ± 0.76	**< 0.001**	0.182	**0.012**
N	3.00	2.96 ± 0.21	3.00	0.294	/	0.428
A	5.93 ± 0.27	5.48 ± 0.85	5.83 ± 0.48	**0.011**	0.676	0.162
L	2.70 ± 0.54	2.30 ± 0.77	2.63 ± 0.65	0.081	0.371	0.223
Abs	1.59 ± 0.69	1.35 ± 0.65	1.67 ± 0.57	0.482	0.990	0.213
DR	4.67 ± 0.62	1.39 ± 1.64	4.04 ± 0.86	**< 0.001**	**0.006**	**< 0.001**
O	5.96 ± 0.19	5.96 ± 0.21	5.88 ± 0.34	0.984	0.285	0.182
AVLT-H						
IR-S	26.19 ± 4.95	18.91 ± 5.52	22.67 ± 4.45	**< 0.001**	**0.017**	**0.012**
SR-S	10.00 ± 1.49	7.09 ± 2.37	8.88 ± 1.54	**< 0.001**	**0.027**	**0.001**
LR-S	9.81 ± 1.98	6.83 ± 2.33	8.33 ± 2.24	**< 0.001**	**0.025**	**0.015**
REC-S	23.63 ± 0.79	22.17 ± 1.80	23.54 ± 0.72	**0.001**	0.562	**0.005**
TMT-A (s)	33.28 ± 10.51	53.23 ± 16.15	42.63 ± 16.08	**< 0.001**	**0.013**	0.055

ESKD, end-stage kidney disease; HCs, healthy controls; ESKD-MCI, ESKD and mild cognitive impairment; ESKD-NCI, ESKD and non-cognitive impairment; MoCA, Montreal Cognitive Assessment; V/E, visuospatial/executive; N, naming; A, attention; L, language; Abs, abstraction; DR, delayed recall; O, orientation; AVLT-H, Auditory Verbal Learning Test-Huashan Version; IR-S, immediate recall total score; SR-S, short-term delayed recall score; LR-S, long-term delayed recall score; REC-S, recognition score; TMT-A, Trail-Making Test, Part A

All quantitative data are expressed as the mean ± standard deviation. Adjusted *p*-values were calculated using one-way analysis of covariance (ANCOVA). Bold figures represent significant differences

### Intergroup differences in GM volumes

**Fig. 3:**
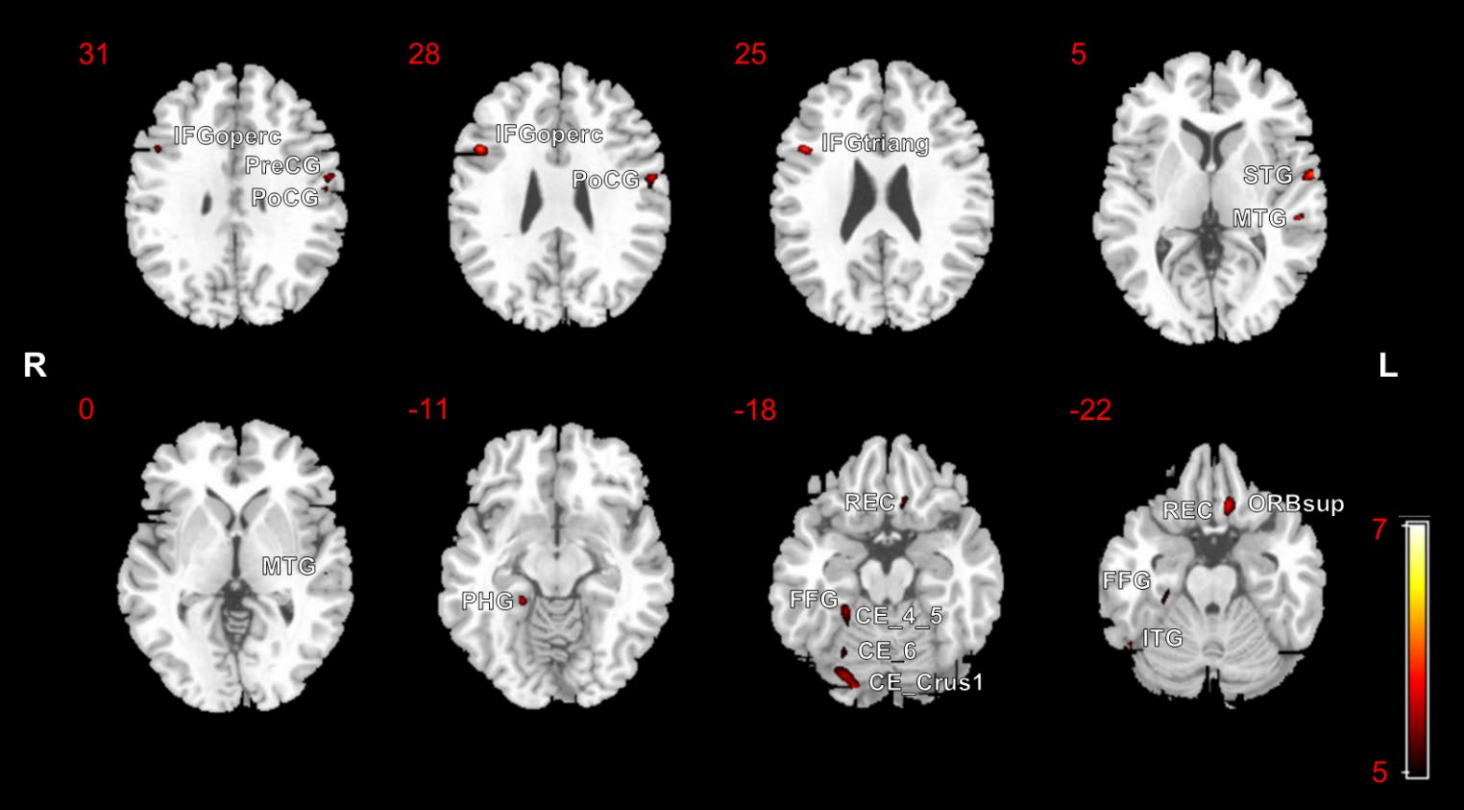
Statistical parametric mapping regions superimposed on a high-resolution T1-weighted scan show decreased grey matter volumes in patients with end-stage kidney disease and mild cognitive impairment, compared to the healthy controls (*p* < 0.05, family-wise error-corrected) R, right; L, left; IFGoperc, opercular part of inferior frontal gyrus; IFGtriang, triangular part of inferior frontal gyrus; PreCG, precentral gyrus; PoCG, postcentral gyrus; STG, superior temporal gyrus; MTG, middle temporal gyrus; PHG, parahippocampal gyrus; REC, gyrus rectus; FFG, fusiform gyrus; CE_Crus1, cerebellum_crus1; CE_4_5, cerebellum_4_5; CE_6, cerebellum_6; ORBsup, orbital part of superior frontal gyrus; ITG, inferior temporal gyrus

Differences in GM volumes between the ESKD-MCI group and HCs are presented in Table 3 and illustrated in Fig. 3. Reduced GM volumes were observed in the right opercular part of the inferior frontal gyrus (IFGoperc), right triangular part of the IFG (IFGtriang), left orbital part of the superior frontal gyrus (ORBsup), left postcentral gyrus (PoCG), left precentral gyrus (PreCG), left superior temporal gyrus (STG), left middle temporal gyrus (MTG), right inferior temporal gyrus (ITG), right fusiform gyrus (FFG), left gyrus rectus (REC), right parahippocampal gyrus (PHG), right cerebellum_crus1 (CE_Crus1), right cerebellum_4_5 (CE_4_5), and right cerebellum_6 (CE_6) in the ESKD-MCI group, compared to HCs (*p* < 0.05 FWE-corrected). No significant increases in GM volumes were observed in the ESKD-MCI group compared to HCs.

**Table 3:** Brain regions with significant grey matter volume reduction in patients with ESKD-MCI vs. HCs, using FWE-corrected maps at *p* < 0.05

Anatomical regions	Brodmann area	Cluster size (voxels)	MNI coordinates (x, y, z)	Peak Z Score	T value	*P* value (cluster level, FWE-corrected
Cluster 1 R IFGoperc R IFGtriang	48	91 67 24	46, 14, 27	5.22	6.17	0.003
Cluster 2 L Superior temporal gyrus L Rolandic operculum	48	40 23 17	-62, -2, 4	5.09	5.96	0.009
Cluster 3 L Middle temporal gyrus L Superior temporal gyrus	22	16 2 14	-54, -28, 4	5.04	5.88	0.020
Cluster 4 R Inferior temporal gyrus R Fusiform gyrus	37	12 11 1	50, -60, -22	4.97	5.78	0.023
Cluster 5 L Gyrus rectus L ORBsup	11	78 38 40	-10, 26, -21	4.97	5.78	0.003
Cluster 6 R Fusiform gyrus R Parahippocampal gyrus R Cerebellum_4_5	30	81 45 17 17	21, -39, -14	4.95	5.74	0.003
Cluster 7 L Postcentral gyrus L Precentral gyrus	43	50 32 18	-58, -4, 28	4.95	5.74	0.007
Cluster 8 R Cerebellum_Crus1 R Fusiform Gyrus R Cerebellum_6	18	121 39 25 55	18, -87, -18	4.94	5.73	0.001
Cluster 9 R Cerebellum_6 R Fusiform gyrus	19	8 6 2	26, -66, -18	4.57	5.19	0.028

ESKD-MCI, end-stage kidney disease and mild cognitive impairment; HCs, healthy controls; FWE, family-wise error; MNI, Montreal Neurological Institute; R, right; L, left; IFGoperc, opercular part of inferior frontal gyrus; IFGtriang, triangular part of inferior frontal gyrus; ORBsup, orbital part of superior frontal gyrus

**Fig. 4:**
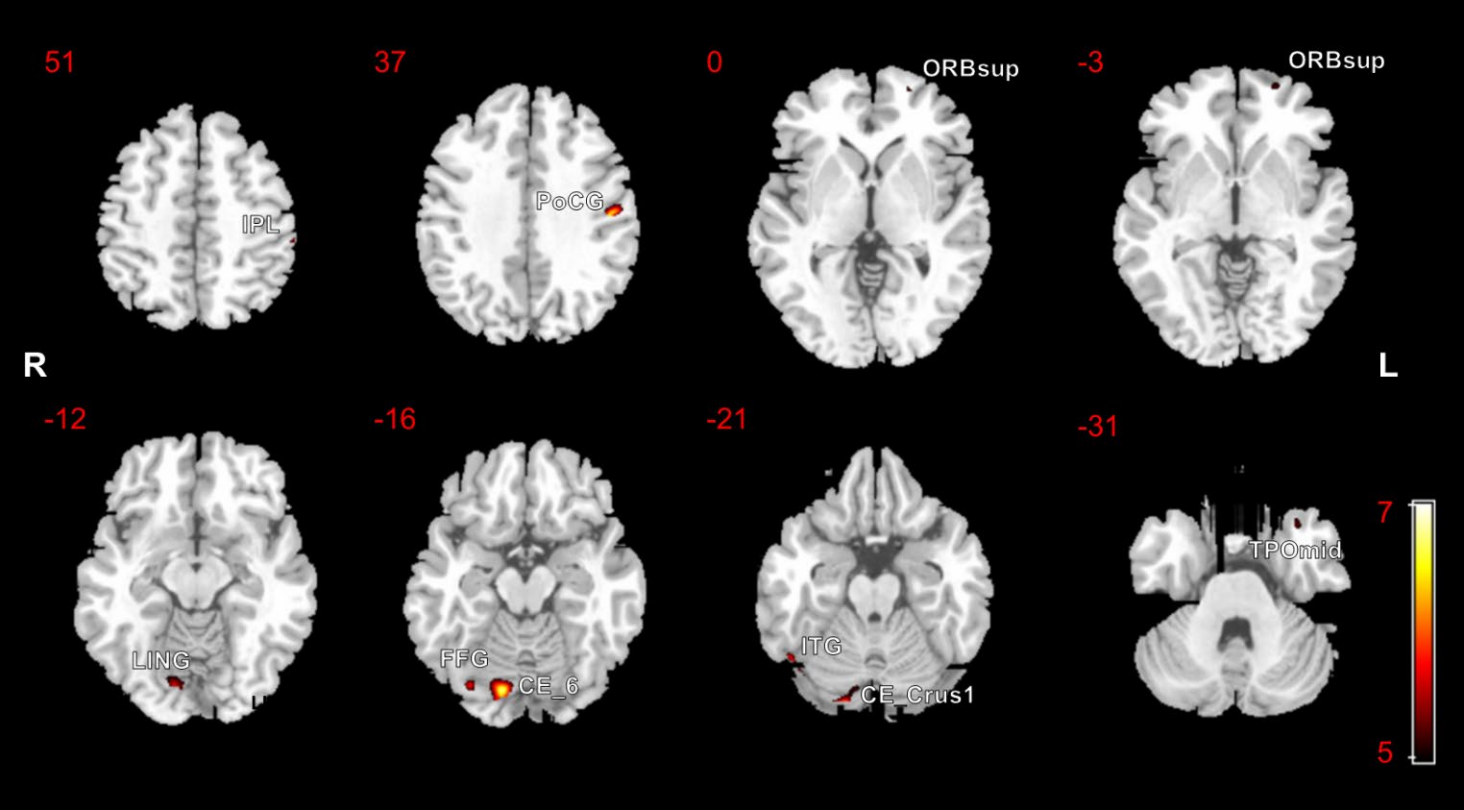
Statistical parametric mapping regions superimposed on a high-resolution T1-weighted scan show decreased grey matter volumes in patients with end-stage kidney disease and non-cognitive impairment, compared to the healthy controls (*p* < 0.05, family-wise error-corrected) R, right; L, left; IPL, inferior parietal but supramarginal and angular gyri; PoCG, postcentral gyrus, ORBsup, orbital part of superior frontal gyrus; FFG, fusiform gyrus; ITG, inferior temporal gyrus; LING, lingual gyrus; CE_6, cerebellum_6; CE_Crus1, cerebellum_crus1; TPOmid, temporal pole: middle temporal gyrus

Differences in GM volumes between the ESKD-NCI group and HCs are shown in Table 4 and depicted in Fig. 4. Decreased GM volumes were observed in the left inferior parietal but supramarginal and angular gyri (IPL), left PoCG, left ORBsup, left temporal pole: MTG (TPOmid), right FFG, right ITG, right lingual gyrus (LING), right CE_Crus1, and right CE_6 in the ESKD-NCI group, compared to HCs (*p* < 0.05 FWE-corrected). No significant increases in GM volumes were found in the ESKD-NCI group compared to HCs.

**Table 4:** Clusters of significant grey matter volume reduction in patients with ESKD-NCI vs. HCs, using FWE-corrected maps at *p* < 0.05

Anatomical regions	Brodmann area	Cluster size (voxels)	MNI coordinates (x, y, z)	Peak Z Score	T value	*P* value (cluster level, FWE-corrected)
Cluster 1 L Postcentral gyrus	3	130 130	-51, -15, 34	5.64	6.82	0.001
Cluster 2 R Cerebellum_6 R Cerebellum_Crus1 R Lingual gyrus	18/17	331 200 65 47	12, -80, -15	5.63	6.80	< 0.001
Cluster 3 R Cerebellum_Crus1 R Inferior temporal gyrus	37	56 26 26	48, -62, -22	5.42	6.45	0.006
Cluster 4 R Fusiform gyrus	19	35 33	32, -76, -15	5.07	5.91	0.011
Cluster 5 L IPL	40	7 7	-57, -32, 52	4.91	5.67	0.030
Cluster 6 L ORBsup	11	18 17	-22, 58, -2	4.72	5.39	0.019
Cluster 7 L TPOmid	20	12 9	-36, 15, -33	4.65	5.30	0.024

ESKD-NCI, end-stage kidney disease and non-cognitive impairment; HCs, healthy controls; FWE, family-wise error; MNI, Montreal Neurological Institute; L, left; R, right; IPL, inferior parietal, but supramarginal and angular gyri; ORBsup, orbital part of superior frontal gyrus; TPOmid, temporal pole of middle temporal gyrus

**Fig. 5:**
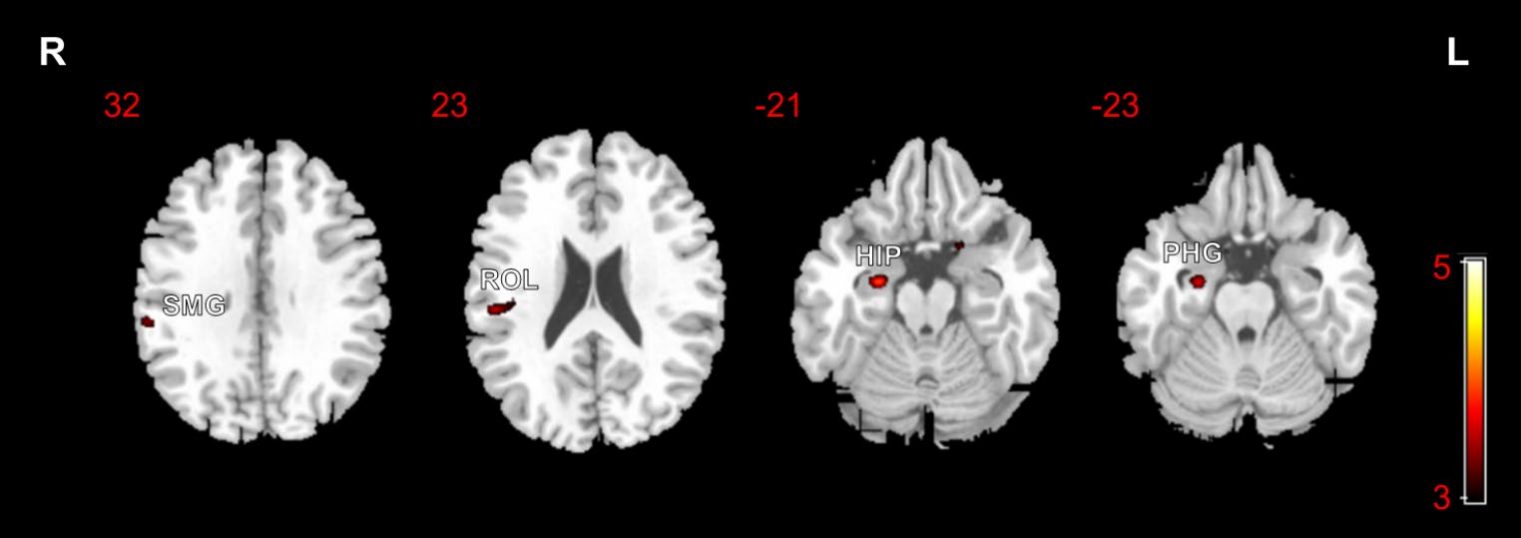
Statistical parametric mapping regions superimposed on a high-resolution T1-weighted scan show decreased grey matter volumes in patients with end-stage kidney disease and mild cognitive impairment, compared to those with end-stage kidney disease and non-cognitive impairment (*p* < 0.001, uncorrected) R, right; L, left; SMG, supramarginal gyrus; ROL, rolandic operculum; HIP, hippocampus; PHG, parahippocampal gyrus

Differences in GM volumes between the ESKD-MCI and ESKD-NCI groups are displayed in Table 5 and visualized in Fig. 5. Decreased GM volumes were observed in the right hippocampus (HIP), right PHG, right rolandic operculum (ROL), and right supramarginal gyrus (SMG) in the ESKD-MCI group, compared to the ESKD-NCI group (*p* < 0.001 uncorrected). No significant increases in GM volumes were found in the ESKD-MCI group compared to the ESKD-NCI group.

**Table 5:** Clusters of significant grey matter volume reduction in patients with ESKD -MCI vs. ESKD-NCI, using uncorrected maps at *p* < 0.001

Anatomical regions	Brodmann area	Cluster size (voxels)	MNI coordinates (x, y, z)	Peak Z Score	T value	*P* value (peak level, uncorrected
Cluster 1 R Hippocampus R Parahippocampal gyrus	35/36	59 39 20	24, -10, -21	3.72	4.08	< 0.001
Cluster 2 R Rolandic operculum R Supramarginal gyrus	48	26 16 8	51, -26, 22	3.30	3.55	< 0.001
Cluster 3 R Supramarginal gyrus	2	12 12	58, -33, 33	3.17	3.39	0.001

ESKD-MCI, end-stage kidney disease and mild cognitive impairment; ESKD-NCI, ESKD and non-cognitive impairment; MNI, Montreal Neurological Institute; R, right

### Association of decreased GM volume with clinical features

Multiple univariate regression analyses demonstrated a negative correlation between the volumes of the right HIP and PHG and the serum calcium level in the ESKD-MCI group, utilizing uncorrected maps at *p* < 0.001 (Table 6). However, no correlation was observed between these brain regions and the cognitive scale tests or other biochemical blood indicators.

**Table 6:** Correlation analysis between decreased grey matter volume with clinical features in patients with ESKD-MCI at a significance level of *p* < 0.001 (uncorrected), and adjusted for age, sex, education level, and TIV.

Anatomical regions	Cluster size (voxels)	MNI coordinates (x, y, z)	Peak Z Score	T value	*P* value (uncorrected)	Clinical features
Cluster R Hippocampus R Parahippocampal gyrus	8 6 2	27, -12, -24	3.41	4.16	< 0.001	Serum calcium

ESKD-MCI, end-stage kidney disease and mild cognitive impairment; TIV, total intracranial volume; MNI, Montreal Neurological Institute; R, right

## Discussion

The findings of the present study suggested that ESKD-MCI results in more impairments in multiple cognitive domains, including global cognition, visuospatial and executive function, and memory, when compared to ESKD-NCI. VBM analysis revealed that both the ESKD-MCI and ESKD-NCI groups presented with diffuse reductions in GM volumes compared to HCs. Notably, the extent of compromised GM was broader in the ESKD-MCI group compared to the ESKD-NCI group. The GM volumes of the right HIP, right PHG, right ROL, and right SMG were lower in the ESKD-MCI than the ESKD-NCI group, using a more liberal uncorrected statistical threshold. Moreover, in the ESKD-MCI group, the volumes of the right HIP and PHG were found to have a negatively correlation with serum calcium levels.

We observed that in comparison to HCs, patients with ESKD-MCI exhibited poorer performance across various cognitive domains, including global cognition, visuospatial and executive function, attention, memory, and processing speed. Compared to patients with ESKD-NCI, patients with ESKD-MCI performed poorly in global cognition, visuospatial and executive function, delayed recall, and immediate recall. These findings align with the hallmark features of MCI, characterized by subtle deficits in one or more crucial cognitive domains (Petersen 2011). While MCI does not interfere with patients’ activities of daily life, it may reduce adherence to complex regimens routinely prescribed to patients with ESKD, and influence the decision-making of ESKD treatment options (Kurella Tamura and Yaffe 2011). Consequently, it becomes imperative for clinicians to heighten their focus on screening for cognitive function among individuals with ESKD. Medical history and neuropsychological examinations are the primary tools used to diagnose MCI. Among the neuropsychological methods, the MoCA stands out as a specifically designed tool for MCI screening (Nasreddine et al. 2005). It is widely used in the auxiliary diagnosis of MCI-related disease entities such as AD-MCI (Julayanont et al. 2014), PD-MCI (Dalrymple-Alford et al. 2010), and hypertension-MCI (Mehra et al. 2020), due to its high sensitivity and superiority over the Mini-Mental State Examination (MMSE) (Pinto et al. 2019). A previous research had indicated that CI, including MCI and dementia, is prevalent among young (21–44 years, approximately 10%) and middle-aged (45–54 years, nearly 20%) hemodialysis patients with ESKD (Kurella Tamura and Yaffe 2011). To mitigate the potential influence of age and dialysis treatment on morphological brain changes, our study deliberately selected young and middle-aged patients with ESKD who had not undergone dialysis. Furthermore, delving into the neurobiological mechanisms underpinning the development of MCI in patients with ESKD would offer valuable insights.

We actually observed that patients with ESKD are not clinically screened for MCI among patients with ESKD, potentially attributed to the lack of effective interventions. A previous study’s findings underscored the significant role played by ESKD-related vascular factors and uraemic neurotoxins in CI (Viggiano et al. 2020b). Although there are no effective drugs for treating MCI, vascular risk factors control and physical and mental activities present promising avenues for patients with ESKD. These strategies can enhance the well-being of patients and caregivers and reduce the risk of poor outcomes (Kurella Tamura and Yaffe 2011; Langa and Levine 2014). Furthermore, our study’s results indicated that patients categorized as having with ESKD-NCI were considered to lack cognitive decline, still differed from HCs in global cognition, memory, and processing speed. These results indicate that patients with ESKD who rely on clinical symptoms and cognitive scales to be divided into groups without CI still may have mild cognitive decline and should be closely monitored.

The VBM analysis revealed that in comparison to HCs, both the ESKD-MCI and ESKD-NCI groups presented a reduction in diffuse GM volumes. As expected, the ESKD-MCI group had a greater extent of brain damage than the ESKD-NCI group. Specifically, the ESKD-MCI group displayed lower GM volume in the right IFG, left ORBsup, left PreCG, left PoCG, left STG, left MTG, left ROL, right ITG, right FFG, left REC, right PHG, and certain right cerebellar regions. These brain regions are implicated in various aspects of cognitive function. For instance, the FFG is primarily responsible for advanced visual functions like face perception and object recognition (Rapcsak 2019). The REC, also called the straight gyrus, situated at the base of the prefrontal cortex, and although its specific function has not yet been elucidated, it may be involved in higher cognitive functioning (e.g., personality) (Selden et al. 1998). The cerebellum plays a role in cognitive and emotional functions, with right-lateralised observed for language and left-lateralised for spatial functions (Schmahmann 2019). Neurodegeneration and direct neuronal injury caused by shared risk factors for the brain and kidney, nephrogenic risk factors, and ESKD treatment-associated risk factors are the primary pathophysiological mechanisms of CI in patients with ESKD (Kurella Tamura and Yaffe 2011). The findings of our study indicate that patients with ESKD-MCI exhibit diffuse structural brain damage that may underlie impairments in multiple cognitive domains.

Previous studies have shown extensive changes in GM volumes alterations among patients with ESKD (Qiu et al. 2014; Li et al. 2018; Wang et al. 2022; Chiu et al. 2019; Gu et al. 2021; Chai et al. 2015), although the results of these studies may vary depending on the sample selection and analytical methodologies. Few studies, however, have stratified the degree of CI among patients with ESKD. Zhang et al. divided patients with ESKD receiving dialysis into minimal nephro-encephalopathy (MNE) and non-nephro-encephalopathy groups, based on their clinical symptoms and attention function (Zhang et al. 2013). This study showed GM volume reduction in the right middle frontal gyrus, right PoCG, right occipital lobe, and left insula in ESRD patients with MNE. These patients receiving dialysis and different evaluation criteria for CI may be the reasons that the results differed from our findings. Recently, a study using diffusion kurtosis imaging found that disrupted brain micro-structures in patients with ESKD-MCI (Zheng et al. 2022). However, this result was based on comparisons between ESKD-MCI and HCs. In the context of our study, it is noteworthy that the observed subtle decrease in GM volume in the right HIP, right PHG, right SMG, and right ROL may signify brain regions in susceptible to damage in patients with ESKD-MCI, in contrast to patients with ESKD-NCI. It is well known that the HIP plays a critical role in episodic memory and navigation (Lisman et al. 2017), and the right HIP is more involved in the memory of locations within the environment (Burgess et al. 2002). An animal study using adult male Sprague − Dawley rats indicated that ESKD can induce cell death in HIP CA1 (Kim et al. 2014). Adjacent to the hippocampus, the PHG is a part of the limbic system. This region plays an important role in memory encoding and retrieval, scene recognition, and social context identification (Squire et al. 2004). The SMG within the inferior parietal lobule plays a role in visual word recognition (Stoeckel et al. 2009). One study showed that the right SMG may also be associated with the maintenance of the ability to recognize emotion (Wada et al. 2021). Therefore, specific damaged regions of the brain in patients with ESKD-MCI may be involved in memory, spatial localisation, visual word recognition, and auditory information processing, which is consistent with the cognitive test results in the present study. The results suggested imaging markers may be provide a relatively objective entry point for the diagnosis of MCI in ESKD patients. Unfortunately, we did not find a direct correlation between these abnormal brain regions and the cognitive scales, possibly due to the small sample size in our study. This underscores the need for larger-scale investigations to unveil potential relationships between structural brain changes and cognitive performance in the context of ESKD-MCI.

Initially, our study did not find significant correlations between the different regions of the brain and clinical measures (*p* < 0.05, FWE-corrected). However, using a more liberal uncorrected threshold (*p* < 0.001), we observed the right HIP and PHG volumes were negatively correlated with serum calcium levels in the ESKD-MCI group. Serum calcium plays critical physiological and biochemical roles in the human body, including neurotransmitter release, signal transduction, muscle contraction, and the electrophysiological stabilisation of cell membranes (Obi et al. 2018), and calcium homeostasis in the human body plays a critical role in many underlying neurological dysfunctions in chronic brain diseases during the aging process (Khachaturian 1994). Excessive calcium entry into the cell leads to an intracellular calcium overload and nerve death. Disturbances in calcium metabolism in patients with ESKD can lead to impaired neuronal Ca^2+^ handling, which in turn causes a series of downstream outcomes, such as synapse loss, amyloid β-peptide accumulation, mitochondrial dysfunction, oxidative stress, and inflammation (Alzheimer's Association Calcium Hypothesis Workgroup 2017), ultimately leading to CI. One study showed that calcium channel blockers have therapeutic effects on CI, due to a variety of causes (López-Arrieta and Birks 2000). These mechanisms include the inhibition of Ca^2+^ overload in neural cells and the blocking of the common channel of neural cell death. The authors hypothesized that the correlation between the decrease in HIP/PHG volumes and increased serum calcium in patients with ESKD-MCI may be due to increased intracellular calcium influx, leading to neuronal death in the HIP/PHG. The findings underscored the significance of maintaining calcium homeostasis as a critical factor in the prevention of CI among patients with ESKD. This insight not only highlights the potential contribution of calcium dysregulation to cognitive decline but also underscores the importance of investigating and optimizing calcium-related interventions to improve CI in this population.

### Limitations

The present study has several limitations. Firstly, it was a cross-sectional study with a small sample size. Sample size expansion and further longitudinal studies are needed to evaluate the changes in brain morphology and CI in patients with ESKD. Additionally, animal studies may better explore the molecular mechanisms of GM volume changes and elucidate their causal relationships. Moreover, the diagnosis of MCI in patients with ESKD requires more comprehensive diagnostic criteria. Therefore, objective and stable imaging diagnostic markers need to be explored further. Incorporating such markers might improve the precision of identifying CI and tracking their progression.

## Conclusion

In summary, the results of the present study indicated that ESKD-MCI was associated with subtle GM volume reductions in several areas of the brain known to be involved in memory, language, and auditory information processing. Notably, serum calcium levels appear to play a role in influencing these subtle morphological alterations. Importantly, even individuals diagnosed with ESKD-NCI, relying on clinical symptoms and cognitive assessments, still shows some cognitive domains impairments. More sensitive imaging methods are needed to explore key brain regions injuries in patients with ESKD-MCI.

## Data Availability

The datasets used and analyzed during the current study are available from the corresponding author on reasonable request.
